# 907. Impact of Antimicrobial Stewardship Prospective Audit and Feedback on Antibiotic Utilization in Hospitalized COVID-19 Patients

**DOI:** 10.1093/ofid/ofac492.752

**Published:** 2022-12-15

**Authors:** Ashfaq Shafiq, Taylor Hori, Selena Pham

**Affiliations:** VA Southern Nevada Healthcare System, North Las Vegas, Nevada; VA Southern Nevada Healthcare System, North Las Vegas, Nevada; VA Southern Nevada Healthcare System, North Las Vegas, Nevada

## Abstract

**Background:**

Studies have consistently shown a high proportion of antimicrobial usage in hospitalized COVID-19 patients despite low prevalence of bacterial co-infection. Improving antibiotic utilization is one of the main goals of antimicrobial stewardship program (ASP). This study aimed to determine the impact of prospective audit and feedback intervention (PAF) performed by ASP pharmacists on antibiotic utilization in hospitalized COVID-19 patients.

**Methods:**

A retrospective chart review was conducted at a 90-bed Veteran Affairs hospital. This study included patients who were admitted to the hospital due to laboratory confirmed COVID-19 infection from November 1, 2020 to January 31, 2021. The primary outcome of this study was to evaluate the impact of ASP PAF on avoiding the unnecessary use of antibiotics in hospitalized patients with acute COVID-19. The secondary outcomes included the prevalence of bacterial co-infection in hospitalized COVID-19 patients and the clinical outcomes.

**Results:**

A total of 199 patients were included in this study. Sixty-one patients (30.7%) had antibiotics started empirically upon hospital admission. Fifty (82%) out of 61 patients had antibiotics discontinued due to the lack of indication for antibiotics during their hospital admission, with 47 (94%) was done by ASP team versus 3 of them (6%) was done by Internal Medicine. Fifty-eight of these patients were followed by ASP team, with 28 patients (48.3%) met SIRS criteria upon admission and 25 (43.1%) with elevated procalcitonin level. The overall readmission rate was 5.8%, with readmission rate of the intervention group to be 6.4% and the non-intervention group to be 5.6% (p = 0.29). The overall mortality rate was 13.1% in hospitalized COVID-19 patients, with 19% reported in the intervention group and 10.1% in the non-intervention group (p = 0.09). Overall, only 5 patients (2.5%) had laboratory-confirmed bacterial co-infection upon admission. Sixteen patients (8%) were found to have microbiologically confirmed nosocomial infection.

**Conclusion:**

The findings suggested that ASP PAF safely and effectively avoided the unnecessary use of antibiotics in hospitalized patients with acute COVID-19 infection. In addition, low prevalence of microbiologically confirmed bacterial co-infection was observed.

**Disclosures:**

**All Authors**: No reported disclosures.

